# Physical and Chemical Properties, Flavor and Organoleptic Characteristics of a Walnut and Purple Rice Fermented Plant Drink

**DOI:** 10.3390/foods13030400

**Published:** 2024-01-26

**Authors:** Hongyu Mu, Tianyi Dai, Si Huang, Kuan Wu, Mingming Wang, Chunlei Tan, Feng Zhang, Jun Sheng, Cunchao Zhao

**Affiliations:** 1 College of Food Science and Technology, Yunnan Agricultural University, Kunming 650201, China; muhongyu0710@163.com (H.M.); dtynongda@163.com (T.D.); 18870918872@163.com (S.H.); wink577@163.com (K.W.); wmm295813@163.com (M.W.); tcl98316@163.com (C.T.); 15762196909@163.com (F.Z.); 2 Yunnan Plateau Characteristic Agricultural Industry Research Institute, Kunming 650201, China; 3 Engineering Research Center of Development and Utilization of Food and Drug Homologous Resources, Ministry of Education, Yunnan Agricultural University, Kunming 650201, China; 4 Key Laboratory of Precision Nutrition and Personalized Food Manufacturing, Ministry of Education, Yunnan Agricultural University, Kunming 650201, China

**Keywords:** walnut and purple rice fermented plant drink, room-temperature yoghurt, rheology, flavor, sensory

## Abstract

In recent years, green and healthy foods have attracted much attention. Plant-based foods have become an alternative to animal-derived foods. In this study, we used walnut and purple rice as the primary raw materials to produce a fermented plant drink. The process included boiling, mixing, grinding, inoculation, fermentation, and sterilization. We then analyzed the similarities and differences between the resulting walnut and purple rice fermented plant drink and an unfermented walnut and purple rice plant drink, as well as dairy-based yoghurt, in terms of physical chemistry, flavor, and sensory characteristics. We also examined the similarities and differences between the walnut and purple rice fermented plant drink and room-temperature yoghurt. The study results revealed that the walnut and purple rice fermented plant drink exhibited greater viscosity than the walnut and purple rice unfermented plant drink and room-temperature yoghurt. Additionally, the former displayed enhanced stability and recovery ability. Notably, distinguishable differences were observed between the three samples in terms of the presence of unknown volatiles and the umami signal, as indicated by electronic nose/tongue and GC-IMS analyses. The umami flavor of the walnut and purple rice fermented plant drink surpasses that of room-temperature yoghurt, while its taste is less salty than that of the walnut and purple rice plant drink. Despite possessing a weaker aroma than dairy-based yogurt, it is more potent than the walnut and purple rice plant drink. Additionally, its relative abundance of olefins, ketones, and alcohols enhances its unique flavor profile, surpassing both other options. Based on sensory analysis, it can be deduced that walnut and purple rice fermented plant drink has the highest overall acceptance rate.

## 1. Introduction

As the global population expands, there will be a significant increase in the demand for food, specifically protein-rich foods [1]. Milk is a nutritious source of protein, calcium, and vitamin D. However, the high levels of saturated fat in milk can adversely affect human health [2]. The escalation in global warming, the increase in chronic diseases, and the mass emergence of vegetarianism and light diets have all played a role in the rise of milk substitutes [3]. Plant-based proteins offer numerous benefits over animal-based sources and are increasingly becoming a groundbreaking and rapidly expanding aspect of various food sectors [4]. Traditional yoghurt is commonly produced using cow’s milk [5], whereas plant-based yoghurt is typically derived from protein-rich plants. Plant-based yoghurt is frequently utilized as a milk alternative owing to its biologically active constituents and the advantages it has for human health [6]. Overall, plants and animals have complementary and symbiotic roles in healthy and sustainable food systems [7].

The walnut, belonging to the Juglandaceae family, has been acknowledged as a nutrient-dense food in various parts of the globe [8]. Walnut kernels contain a high concentration of protein, fat, vitamins, minerals, fiber, and polyphenolic compounds [9]. Containing unsaturated fats and polyphenols, they offer a myriad of health benefits, including reducing the risk of cardiovascular and coronary heart disease, treating type II diabetes, preventing and treating specific cancers, and alleviating symptoms associated with aging and neurological diseases [10]. Purple rice is a glutinous rice variety that is highly valued as a colored rice germplasm resource in China due to its long history of cultivation [11]. Research has demonstrated that the nutritional elements of colored rice, including protein, amino acids, and minerals, surpass those of regular white rice [12]. In addition, purple rice is also rich in polyphenols, flavonoids, anthocyanins, dietary fiber, vitamins, and essential minerals such as Fe, Zn, Ca, and P. These nutrients have been proven to be beneficial for human health [13]. Anthocyanins, the major components of pigmented rice, exhibit a range of biological activities, including free radical scavenging, antioxidation, anti-tumor, anti-atherosclerosis, hypoglycemic, and anti-allergic effects [14]. These activities are vital for preventing diseases and promoting health, making pigmented rice an attractive candidate for food and pharmaceutical applications [15]. Walnuts and purple rice are high-quality plant-based foods with significant nutritional and medicinal benefits. They contain numerous bioactive substances, including phenolic compounds, unsaturated fatty acids, and anthocyanins, which are beneficial to human health. The combination of walnuts and purple rice not only enriches nutrition but also enhances their unique flavors, making it an excellent choice for dairy-free milk alternatives.

Compared to traditional animal-based milk yoghurt, plant-based fermented drinks are from safe sources and have the benefits of no cholesterol, no trans fats, no lactose, and no or low levels of allergens and fiber, which are beneficial for cardiovascular health, weight management, and managing lactose intolerance and cow’s milk allergy [16]. Plant-based fermented beverages offer unique nutritional benefits and excellent product safety, giving them great potential for market development. A growing body of research also shows that gentle fermentation can improve the taste and flavor of plant-based products [17,18]. The aim of this study was to investigate the effect of mild fermentation with *Lactobacillus plantarum* on the flavor and sensory properties of a walnut and purple rice plant fermented beverage and to further analyze the similarities and differences between the walnut and purple rice fermented plant drink and ordinary room-temperature yoghurt, in order to provide a theoretical basis for the development of alternatives to animal-sourced yoghurt.

## 2. Materials and Methods

### 2.1. Raw Materials

Hulled walnut kernels, purple rice, fresh milk, sucrose, carboxymethylcellulose CMC, modified starch, and rapeseed oil were purchased from a local market (Kunming, China). *Lactobacillus plantarum* (GDMCC accession number GDMCC1.2685) was purchased from Guangdong Provincial Microbial Strain Preservation Centre (Guangzhou, China). Direct-pitch commercial fermenters (Mild1.0 comprising *Lactobacillus bulgaricus* and *Streptococcus thermophilus*) were purchased from Chr. Hansen, Denmark (Chr. Hansen, Hersholm, Denmark). All other chemicals used were of analytical grade and are commercially available.

### 2.2. Strains and Growth Conditions

First, activated *Lactobacillus plantarum* was inoculated in 100 mL MRS broth medium, inoculated in 2% (*v*/*v*) inoculum, and incubated at 37 °C for 16 h to obtain starter cultures for fermentation. Afterwards, the concentration of *Lactobacillus plantarum* was adjusted to 1 × 10^8^ CFU/mL and then centrifuged at 5000 rpm for 5 min at 4 °C, washed three times with sterile saline, and recovered as a seed solution for fermentation.

### 2.3. Sample Preparation

The method was slightly adjusted according to the existing formula and process in the laboratory.

Walnut and purple rice fermented plant drink: After washing, purple rice was soaked in deionized water for 2 h. After soaking, the purple rice was heated at 95 °C for 30 min, and an equal amount of peeled walnut kernels were added and then ground with deionized water (80 °C) at a ratio of 1:3. The walnut–purple rice milk was finely ground in a colloid mill (JM-F80, Shanghai Toppay Machinery Equipment Co., Ltd., Shanghai, China) for 15 min until there were no obvious large particles and the texture was fine and uniform. The 8.5% sucrose, 0.3% CMC, and 0.5% rapeseed oil were weighed and added to the walnut and purple rice milk. The mixture was stirred with a blender, heated at 95 °C for 1.5 h, cooled to 34 °C, and inoculated with 1% (*v*/*v*) culture. The walnut and purple rice fermented plant drink (H1) was fermented at 34 °C for 14 h. The fermentation was stopped when the pH reached about 4.5, and the milk was sterilized at 85 °C for 30 min and stored at room temperature.

Walnut and purple rice plant drink: Using the same process and formula, the unfermented walnut and purple rice plant drink (H2) was used as the control group and stored at room temperature.

Room-temperature yogurt: The milk was preheated to 45 °C, and 8.5% sucrose, 2% modified starch and 0.6% complex thickening emulsifier HBT-A8570 were added and heated to 65 °C for 15 min. The milk was then homogenized using a high-pressure homogenizer (GYB60-6S, High-Pressure Homogenizer, Shanghai Donghua High-Pressure Homogenizer Factory, Shanghai, China) at 25 MPa for 5 min. The milk was then pasteurized in a six-coupled electric thermostatic mixer (JJ-6A-6H, Changzhou Jibori Instrument Manufacturing Co., Ltd., Changzhou, China) for 2 min. The sterilized mixed milk was cooled to 37 °C and inoculated with 0.5‰ of the commercial direct pitch fermenter. Fermentation was stopped at 42 °C for 6 h to reach a pH of approximately 4.5, and then the milk was sterilized at 85 °C for 30 min to obtain room-temperature yoghurt (C1). It was stored at room temperature, and each treatment was repeated in triplicate.

### 2.4. Physicochemical Analysis 

Titration acidity (TAA): Samples were prepared by diluting (10 g) with distilled water (20 mL). Phenolphthalein solution (2 mL, 5% *w*/*v*) was added to the diluent as an indicator. The mixture was titrated with 0.1 M NaOH to express a pink color that did not fade within 30 s. TAA (°T) was calculated as the volume of NaOH consumed (mL) multiplied by ten.

pH: After the samples were brought back to room temperature, they were stirred uniformly with a glass rod. The pH of the fermented milk was measured using a pH meter (pHS-3C, Shanghai Yitian Scientific Instrument Co., Ltd., Shanghai, China), and the samples were repeated three times.

The samples were analyzed for their chemical composition (protein, fat, total solids, and ash) using standard AOAC 2005 procedures [19,20].

### 2.5. Rheological Analyses

The samples were characterized for their rheological behavior according to the previous study conditions [21,22,23,24]. The rheological properties of the samples were analyzed using an MCR 302 rheometer, Anton Paar Austria, Graz, Austria) equipped with a temperature control system. A 50 mm diameter parallel plate configuration with an interplate gap of 1 mm was used.

Relationship between strain and time: The temperature of the rheometer was set at 25 °C, 10 Pa shear stress was applied to the sample, and the change in strain with time was recorded for 5 min. A total of 50 points were taken for data detection. Time was taken as the horizontal axis and shear stress as the vertical axis.

Relationship between apparent viscosity and shear rate: The samples were poured between the rheometer plates and placed at room temperature for 15 min. The test was carried out with a CC25 DIN fixture, and the test distance was adjusted to 500 μm. The shear rate was increased from 0.01 S^−1^ to 1000 S^−1^, and 50 points were taken for data detection. The shear rate was used as the abscissa, and the apparent viscosity was used as the ordinate to draw the curve.

Determination of dynamic rheological properties: The detection temperature was set at 25 °C, the shear strain was 0.5%, the frequency was increased from 0.1 to 10 Hz, and the duration was 10 min. The changes in the elastic modulus (G′) and viscous modulus (G″) of the two starch pastes with the increase in frequency were measured.

Relationship between shear rate and shear stress: The samples were poured between the rheometer plates and allowed to stand at room temperature for 15 min. The test was carried out with a CC25 DIN fixture, and the test distance was adjusted to 500 μm. The shear rate increased from 0.01 S^−1^ to 1000 S^−1^, from which 50 points were taken for data detection, and then decreased from 1000 S^−1^ to 0.01 S^−1^ for data detection. Taking the shear rate as the abscissa and the shear stress as the ordinate, the curve was drawn.

### 2.6. Electronic Nose and Electronic Tongue Determination

Electronic nose: 7 g of sample was weighed into a 30 mL headspace vial and detected using an electronic nose (cNose, Shanghai Baosheng Industrial Development Co., Ltd., Shanghai, China) with the following parameters: incubation at 50 °C for 20 min, an oscillation speed of 500 r/min, an injection volume of 5000 μL, an injection speed of 125/s, an inlet temperature of 200 °C, a pressure of 10 kPa, a flow rate of 30 mL/min, and an injection time of 45 s. Each sample was replicated three times.

Electronic tongue: 100 g of sample was weighed, and 100 mL of purified water was added. The water was stirred to mix the water and yoghurt samples thoroughly and evenly, and centrifugation was carried out at 3000 rpm for 10 min before removing the supernatant and testing with an electronic tongue instrument (Ctongue, Shanghai Bao Sheng Industrial Development Co., Ltd., Shanghai, China). Each group of samples was tested three times in parallel at room temperature.

### 2.7. HS-GC-IMS Volatile Flavor Compound Analysis 

A gas chromatography–ion mobility spectrometer (FlavourSpec 1 H1-100053), GAS, Dortmund, Germany), with an Rtx-Wax column (30 m × 0.53 mm, 1 μm) from Restek, Bellefonte, PA, USA and an automated headspace injection unit (Combi PAL) from CTC, Zwingen, Switzerland, was used in this study.

Headspace sampling was used, and the sampling conditions were optimized. The optimized headspace sampling conditions were as follows: 0.5 mL of the sample was sealed in a 20 mL headspace vial and heated and shaken at 70 °C for 15 min, and then 200 μL of the sample was taken from the upper space of the headspace vial for detection. The temperature of the headspace injection needle was 85 °C. The temperature of the headspace injection needle was 85 °C. The GC-IMS test conditions were set as follows: the temperature of the GC column was 60 °C, and the temperature of the drift tube was 45 °C. The flow rate of the carrier gas for GC-IMS was 2 mL/min for 0~2 min, 2~10 min, 2~10 mL/min for 2~10 min, 10~90 mL/min for 10~30 min, and 90 mL/min for 30~40 min. The flow rate of the drift gas for ion mobility spectrometry (IMS) was kept constant at 150 mL/min. The carrier gas and the drift gas were both nitrogen (purity: 99.999%). Each sample was replicated 3 times.

### 2.8. Sensory Analysis 

Sensory analysis of the samples was performed by a group of experienced sensory testers (*n* = 12, age range 20–59) recruited from the School of Food Science and Technology, Yunnan Agricultural University. The team members were asked to rate the intensity at a level of 0 to 10 for taste and flavor descriptors. Team members were also asked to rate the overall acceptability of the samples at a level of 0 to 10. The questionnaire includes the definition of descriptors for sensory testing, which can be found in Table 1. Sensory analysis was carried out in the sensory analysis room and in duplicate within two days.

### 2.9. Data Analysis

SPSS version 23 (IBM SPSS Inc., Chicago, IL, USA) was used to conduct a one-way analysis of variance (ANOVA) with Tukey’s test at a 95% confidence level. Values are expressed as the mean ± standard deviation. PCA and PLS-DA were performed using OriginPro 8.6 software (OriginLab Corporation, Northampton, MA, USA) and SIMCA 14.1 software (Biometric Software Developer Umetrics, Umeå, Sweden), respectively.

## 3. Results and Discussion 

### 3.1. Physicochemical Analysis

Table 2 shows the comparison of the physical and chemical components of the three samples. The results showed that the contents of protein, fat, and solids in the walnut and purple rice fermented plant drink were higher than those in ordinary milk-based yoghurt (*p* < 0.05). Acidity is a very important indicator of yoghurt fermentation. An acidity of 4.5 is usually used as the end point of fermentation in yoghurt production [25]. The pH of unfermented H2 was 6.38 and was reduced to 4.45 (H1) after incubation with a mixture of *Lactobacillus plantarum* fermentation broth at 34 °C for 14 h, confirming the effectiveness of the fermentation process. Acidity and pH were mainly influenced by lactic acid produced by the growth of lactic acid bacteria [26]. The pH of dairy yoghurt is usually around 4.5, and the pH range in the dairy and non-dairy samples is 3.4 and 4.4. The total titratable acidity showed significant differences between dairy and plant samples. Compared to the walnut and purple rice fermented plant drink, the TTA of the dairy yoghurt was significantly higher, and the difference between the two was about 30. Because walnut and purple rice contain a large amount of fiber and other substances, the total solids content of walnut–purple rice yoghurt is much higher than that of ordinary dairy yoghurt. Walnut and purple rice yoghurt with a high fiber content enhances the effects of intestinal peristalsis and improves digestive function [27].

### 3.2. Rheological Measurements

**Figure 1:**
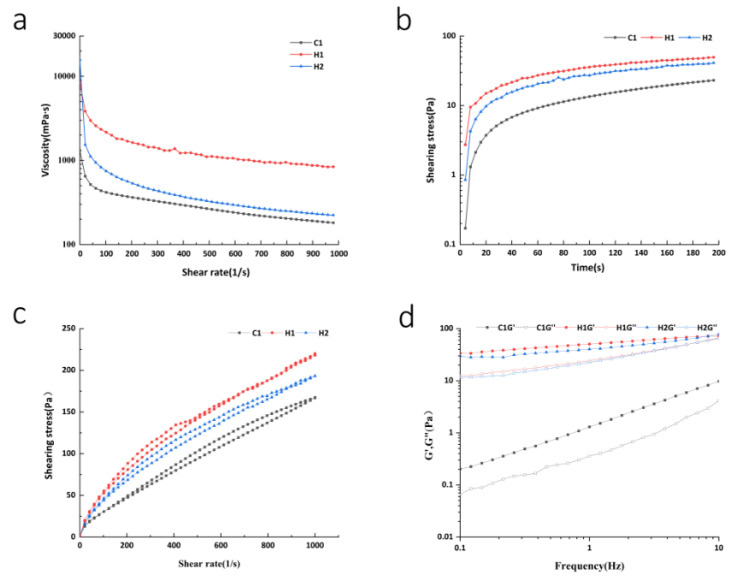
(**a**) Relationship between viscosity and shear rate; (**b**) relationship curve between time and shear stress; (**c**) the relationship curve between shear rate and shear stress; (**d**) viscoelastic characteristic curve.

Rheological properties can indicate changes in viscosity as the shear rate varies. Viscosity is a measure of a liquid’s physical characteristics that resist flow under shearing conditions, which can help to elucidate the attributes of dairy products [28]. Newtonian fluid is an ideal fluid whose viscosity remains unaffected by the shear rate. On the contrary, non-Newtonian fluids do not exhibit this ideal flow property [29]. Figure 1a,b display the relationship between viscosity, shear rate, and shear time for all sample groups. The results indicate that increasing shear rate and time led to a rapid decrease in apparent viscosity for each group before stabilizing gradually. This observation revealed the characteristics of high pseudoplasticity and the shear-thinning phenomenon of non-Newtonian fluid [30]. When the rate of shear is low, the damage level to the gel network structure inside the sample is minimal. Subsequently, as time and the rate of shear increase, the level of damage to the gel network structure inside the sample increases until it is completely destroyed. Consequently, electrostatic interaction and hydrophobic interaction weaken, ultimately resulting in the phenomenon of shear thinning [31]. The viscosity of normal-temperature yoghurt is lower than that of the walnut and purple rice fermented plant drink, suggesting that despite the addition of stabilizer and modified starch to normal-temperature yoghurt, the higher total solids content of the walnut and purple rice fermented plant drink results in more binding force between the particles, leading to higher viscosity. Additionally, fermentation promotes the precipitation of polysaccharides and other substances. Compacted polysaccharides are capable of modifying the structural network via non-covalent interactions and of incentivizing thickening behavior. This leads to an increase in the viscosity of the sample system and a decrease in its fluidity [32]. The shear stress and shear stress values of the walnut and purple rice fermented plant drinks were higher than those of the room-temperature yoghurt samples among the three groups of samples, indicating that the overall viscosity of the former was higher. The shear stress and shear rate relationship curve of various samples can be seen in Figure 1c. The walnut and purple rice fermented plant drink yields somewhat higher shear stress than the regular dairy yoghurt and walnut and purple rice plant drink. When an external force is exerted, there is a significant alteration in the structural composition of the walnut and purple rice fermented plant drink. Meanwhile, the thixotropic ring area of this yoghurt is smaller as compared to the other two groups, which suggests that its structural composition remains largely unaffected upon the application of external force. After the changes in the organizational state, the energy needed to revert to the initial state is notably lower in the walnut and purple rice fermented plant drink group compared to the other two groups. This suggests that this group is comparatively more stable and exhibits a more robust recovery ability. Prior research has demonstrated that yogurt with fish gelatin produces higher shear stress than control yogurt lacking gelatin, resulting in a stronger gel structure, making the system more stable [33].

The viscoelastic features of yoghurt specimens can be analyzed further by conducting dynamic oscillation trials. Figure 1d demonstrates that upon increasing the frequency, the G′ and G″ values of yoghurt samples increased. Furthermore, for the same type of samples, G′ was found to be greater than G″. This indicates that the specimens exhibit a certain stiffness (elastic behavior), presenting a semi-solid weak gel structure in line with the characteristics of yoghurt [34]. The G′ and G″ measurements of walnut and purple rice yoghurt were higher than those of ordinary room-temperature yoghurt, implying that walnut and purple rice yoghurt exhibit more solid behavior than ordinary room-temperature yoghurt. The starch particle structure in the walnut and purple rice fermented plant drink was chain-like and dispersed, with a strong interaction between particles (gel formation). Consequently, the gel of the walnut and purple rice fermented plant drink was softer than that of ordinary room-temperature yoghurt [35]. Additionally, the storage modulus (G′) and loss modulus (G″) of the walnut purple plant drink were lower compared to those of the stirred walnut and purple rice yoghurt, demonstrating that the structure of the latter was denser. This further supports the notion that fermentation enhances the gelling properties and robustness of the walnut and purple rice fermented plant drink [36].

### 3.3. Analysis of Electronic Tongue and Electronic Nose

#### 3.3.1. Electronic Tongue Analysis

**Figure 2:**
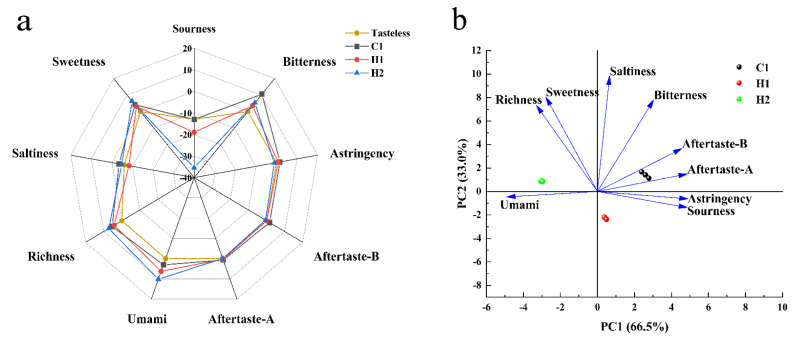
Radar (**a**) and PCA (**b**) of the electronic tongue’s assessment of different samples. Note: The “tasteless point” is the output of the reference solution, which comprises KCL and tartaric acid. The sour taste tasteless point is −13, the salty taste tasteless point is −6, and the tasteless points of other indicators are 0. Consequently, when the sample’s taste value is lower than the “tasteless point”, the sample is considered to have no taste, and vice versa. The table’s richness represents the aftertaste of the umami, indicating the persistence of the umami in the sample, called the umami persistence. The bitterness aftertaste reveals the degree of bitterness residue, and the astringent aftertaste indicates the degree of astringency residue.

An electronic tongue is a bionic taste analysis device capable of objectively measuring food taste changes through alterations in the lipid membrane potential of an artificial taste sensor array [37]. The flavor radar images for various samples were acquired by extracting the response values from each sensor, as illustrated in Figure 2. Figure 2a shows that the acidity values for the three samples are not significant. Their acidity is lower than or proximate to the taste threshold. Additionally, other taste indicators are present above the taste threshold, substantiating their effectiveness as taste markers. H2 has a lower degree of astringency, bitterness aftertaste, and astringency aftertaste than the taste threshold. Acidity and sweetness are significant indicators of flavor across the samples. Differences between the samples’ acidity and sweetness are also readily apparent. The study clearly demonstrates the distinct sweetness attributes of all three samples. The data indicate that ordinary room-temperature yoghurt possesses the strongest sour taste, approaching the threshold of tastelessness, while the walnut and purple rice yoghurt exhibits considerably less sourness compared to the plain yoghurt (a numerical difference of several units on a standard scale) The sour taste of the walnut and purple rice plant drink is significantly lesser than that of room-temperature yoghurt. Additionally, the sweetness of the walnut and purple rice plant drink is greater than that of normal dairy-based yoghurt, with the walnut and purple rice fermented plant drink exhibiting the least amount of sweetness. The study results revealed higher bitterness, bitter aftertaste, astringency, and astringency aftertaste scores in yoghurts as compared to odorless points, except for the walnut and purple rice plant drink Additionally, technical term abbreviations should be explained upon their first usage. It is important to note that the terms ‘bitterness’ and ‘astringency’ do not imply that the dairy products tasted extremely bitter. Instead, these terms reflect the richness, milk flavor, and strong sense of taste in these products. For the walnut and purple rice fermented plant drink, it is essential to consider the bitter taste in the results, as walnut contains polyphenols and other compounds. It is crucial to account for the sensory situation of the raw material itself. The bitterness and astringency of the other two samples are lower than those of ordinary yogurt at room temperature. In particular, there is a notable difference in bitterness between the three samples, while the difference in astringency and aftertaste is minor. Richness refers to the aftertaste of umami, reflecting the persistence of umami, which can also be known as umami persistence. The study’s findings reveal that the sample’s richness is greater than its umami. This is because in calcium-rich samples such as yoghurt and cheese, the output of umami is inhibited by calcium, while its effect on aftertaste is unaffected The instrument test output indicates a higher aftertaste value than umami value, which aligns with the senses being unaware of the umami taste while perceiving the richness. The umami test revealed significant differences in umami, richness, and saltiness between the three yoghurts, with the dairy yoghurt having the lowest umami and richness and the highest saltiness. The umami of the walnut and purple rice fermented plant drink sample H1 is superior to that of C1, albeit less rich and with the lowest saltiness score. Sample H2 possesses the highest umami and richness, with a substantial saltiness score as well.

Taste is a complex sensation perceived by the taste buds, making it narrow-minded to assess a specific taste in isolation. As such, we conducted a PCA analysis on the taste data from the three samples (refer to Figure 2). PCA, a widely used statistical analysis method, was employed [38]. The first and second principal components were used as the horizontal and vertical axes, respectively, with variance contribution rates of 70.2% and 29.8%, respectively. Following analysis of all taste indices in this test, cluster analysis was performed on the three yoghurt samples. The graph clearly indicates that the three samples display distinct taste variations and unique characteristics. There are significant variances in the first and second principal components of the three samples. Figure 2b depicts that the first principal component is mainly impacted by sourness, bitterness, and umami, while the second principal component is influenced by saltiness, sweetness, richness, and bitterness. The variations in flavor profiles of the three yoghurt samples are largely discernible through these aspects.

#### 3.3.2. Electronic Nose Analysis

**Figure 3:**
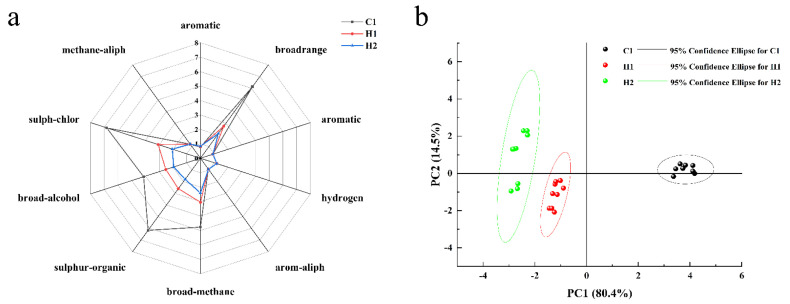
Radar (**a**) and PCA (**b**) of electronic noses’ assessment of different samples.

The electronic nose and electronic tongue work in a similar way [39]. The aforementioned bionic olfactory analysis technology is capable of performing a comprehensive evaluation of volatile odor information within samples in a rapid, sensitive, and non-destructive manner [40]. The radar chart, which displays the average of the results from three tests conducted on the 10 sensors of the electronic nose at 70 s, is illustrated in Figure 1a. Figure 3 reveals that the electronic nose has a substantial response to the three sample groups. The scent of standard yoghurt at room temperature is powerful, with the electronic nose exhibiting a significant response. Additionally, standard yoghurt, when sealed and left stationary, accumulates a relatively high concentration of headspace. In contrast, the aroma of the walnut and purple rice plant drink is faint, and the concentration of enrichment during standing is minimal. The radar map indicates that sensors No. 2, No. 6, No. 7, No. 8, and No. 9 respond to the three samples. Sensor No. 2 is sensitive to small molecule nitrogen oxide gases, No. 6 is sensitive to short-chain alkanes such as methane, No. 7 is sensitive to inorganic sulfur gases, No. 8 is sensitive to alcohol ether aldehyde ketones, and No. 9 is sensitive to organic sulfur gases. Nonetheless, considerable variations are present in the response values of these sensors. Throughout the testing process, the C1 sensors for normal-temperature yoghurt showed the highest response values, with particular sensitivity found in sensors 2 (to small molecule nitrogen oxide gas), 7 (to inorganic sulfur gas), and 9 (to organic sulfur gas). Additionally, the volatile odor of the walnut and purple rice fermented plant drink was lower than that produced by C1; the response value from sensor H2 was especially lower than that of C1. The order of the three smells is C1 > H1 > H2. H1’s response value was significantly greater than that of H2, even though most raw materials were identical. The key factor at play was lactobacillus fermentation, which stimulated the release of related compounds and led to the improvement in the response value [41].

According to the principal component analysis (PCA), the combined contribution rate of the first and second principal components amounts to almost 95%, effectively encompassing the majority of the sample’s original information. Notably, the contribution rate of the first principal component is 80.4%, while that of the second is 14.5%. The scatter plots of the samples from the three groups clustered together, demonstrating good repeatability within each group and high similarity of the sample data. Furthermore, there was a clear distinction between the groups, as revealed by the mutual clustering. The electronic nose effectively discerned differences between the three samples. The normal room-temperature yoghurt exhibited the largest discrepancy in odor compared to the other two samples, as indicated by the sensor readings and depicted in the figure. Furthermore, the yoghurt was significantly distinct from the other two. On the X and Y axes, the walnut and purple rice plant drink was located at a lower position, which suggests that its volatile odor is comparatively weaker than that of the other two samples.

### 3.4. GC-IMS Analysis of Volatile Flavor Compounds

#### 3.4.1. Analysis of Spectrum of Volatile Flavor Components 

**Figure 4:**
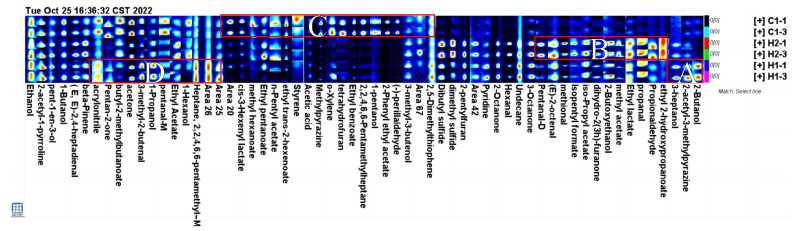
Gallery plot of samples. Note: The graph displays all the signal peaks chosen in a sample, with each column showing the signal peaks of a specific volatile organic compound in diverse samples. Certain substances have the abbreviations m and -D, referring to the monomer and dimer of the same element. The numerical value represents an unidentified peak. The parts of the different samples with higher content were classified and labelled with the letters A–D.

The fingerprint spectrum was reconstructed from all the peaks in the GC-IMS spectrum using the Gallery Plot plug-in, and the characteristic peak area was identified [42]. Figure 4 displays the VOC fingerprints of various samples. The horizontal axis presents the selected characteristic identification peak, and each column corresponds to a VOC. The vertical axis denotes the sample identification numbers, and each row represents a sample. The background is colored black and blue, whereas the red color indicates higher content, and the lighter color shows lower content [43]. The table displays the variation in VOC content across different samples. Each row represents the type of VOC contained in the sample, while each column shows the content variation. To aid observation and comparison, substances with similar variation rules are grouped and compared. These groups are then divided into four regions—A, B, C, and D. The A and D areas of the map exhibit the distinctive volatile components of the walnut and purple rice fermented plant drink H1. The main characteristics include acrylonitrile, 2-butanol, 2,2,4,6,6-pentamethylheptane-M, 2-acetyl-3-methylpyrazine, and 3-heptanol. The B area (shown in the diagram) indicates the distinct volatile compounds of H2, such as ethyl trans-2-hexenoate, propionaldehyde, 1-propanol, ethyl pentanoate, (E)-2-octenal, and isopentyl formate. The substances depicted in diagram b represent the specific volatiles of H2, mostly esters, with a concentration significantly higher than in other samples. It is worth noting that technical abbreviations are explained on their first use to ensure comprehension. The C region in the diagram includes volatile substances such as (−)-perillaldehyde, 2-phenylethyl acetate, methylpyrazine, 1-propanol, and ethyl benzoate, which are characteristic of C1. The characteristic volatile substances of C1 are represented by the peak colors, which are significantly darker in the three samples. This indicates a higher content of these substances than in the other samples. Objective evaluation is prioritized, and abbreviations of technical terms are explained.

#### 3.4.2. Analysis of Relative Contents of Volatile Substances

According to the examination of GC-IMS outcomes, there is a positive correlation between the characteristic peak intensity of an aroma substance and its content in the sample. The study identified a total of 55 chemical compounds, consisting of 5 ketones, 10 alcohols, 9 aldehydes, 13 esters, 2 acids, 2 ethers, 8 olefins, and 8 heterocyclic compounds. Among these compounds, ketones, alcohols, esters, and aldehydes were the primary volatile compounds of both samples. Relative content of different substances analysed through Table 3, we identified 21 significant flavor compounds (with a relative content greater than 1%) in the three samples. These compounds enhance the overall flavor of the product.

**Figure 5:**
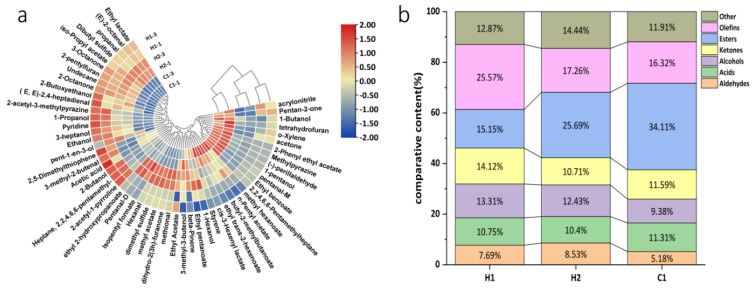
Cluster heat map (**a**) and difference map (**b**) of relative content of volatile flavor substances in samples. Note: in the clustered heatmap, red represents relatively high levels and blue relatively low levels, with darker colors representing greater differences.

To enhance characterization of varying volatile compounds, we calculated the relative difference among the flavor compounds in the three samples based on signal intensity on the fingerprint, demonstrated in Figure 5. The similarity of the acquired VOC fingerprints was assessed using statistical approaches like the heat map and clustering method, as detailed in Figure 5a. The volatile organic compounds (VOCs) present in three samples were analyzed and vertically clustered. As can be seen in Figure 5b, there are some differences between the contents of the relative characteristic volatile classifications of H1, H2, and C1 in the three samples. From the figure, it is apparent that the primary components of the walnut and purple rice fermented plant drink are H1 (with 25.57% olefins, 15.15% esters, 14.12% ketones, and 13.31% alcohols), followed by H2 (with 25.69% esters, 17.26% olefins, 12.43% alcohols, and 10.71% ketones) and C1 (with 34%, 11% olefins, 16.32% alcohols, and 11.31%). The content of 2-acetyl-1-pyrroline was the highest in this product, and the threshold for this compound was lower, which gives it a savory taste and contributes to the overall flavor [44]. Compared to H2, the proportion of olefins in walnut and purple rice yoghurt H1 significantly increased, with a concurrent increase in ketone content. The ketones mainly resulted from microbial metabolism during fermentation [45]. They are produced mainly by the oxidation of esters and unsaturated fatty acids [46]. Many of these ingredients have a pleasant aroma, possibly accounting for the reduction in relative ester levels in H1 walnut and purple rice fermented plant drink, and the majority of ketones had a high threshold and did not significantly affect the flavor profile [47]. Fermentation can alter the composition and proportions of volatile flavor compounds, leading to a more intense flavor profile. A combination of flavor compounds give rise to the characteristic flavor of the walnut and purple rice fermented plant drink H1. In comparison to regular temperature yoghurt C1, esters exhibit the greatest disparity in their relative content between the two. The relative content of esters in plain normal-temperature yoghurt C1 is twice as high as in H1 yoghurt. Esters are key components of food flavor As well as giving foods a special “floral and fruity flavor”, they can also mask the “unpleasant, irritating taste” caused by free fatty acids [48]. Aldehydes primarily arise from the oxidation of lipids [49]. The threshold is generally low, with a fragrant and fruity taste, which also gives H1 a different flavor from ordinary yoghurt C1 at room temperature.

#### 3.4.3. Principal Component Analysis of Volatile Flavor Compounds

**Figure 6:**
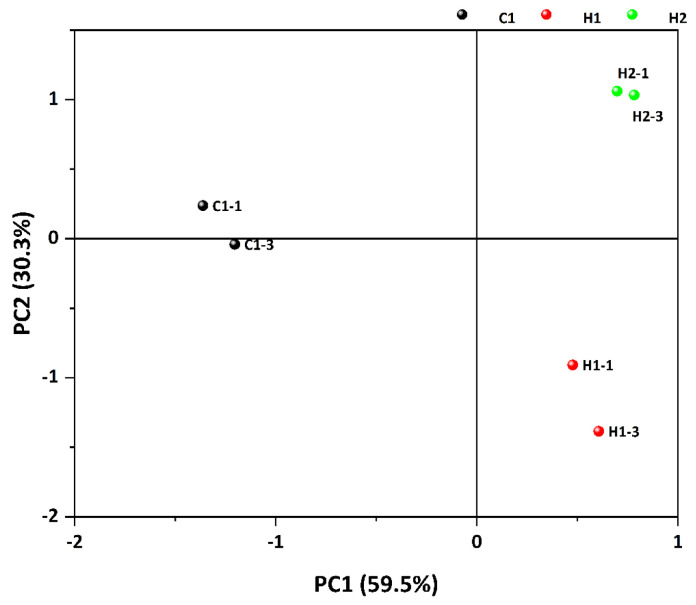
PCA analysis diagram of volatile substances in three samples.

Principal component analysis (PCA) is a multivariate statistical approach that diminishes the dimensions of a dataset composed of numerous related variables, at the same time preserving as much variation within the data as possible This method has garnered broad usage in investigating variations in food aroma compounds [50]. Principal component analysis was conducted on the peak intensities of the volatile compounds in the three specimens, and Figure 6 displays the findings. The first principal component PC1 made a contribution rate of 59.5%, while the second principal component PC2 contributed 30.3%. The combined contribution rate of the first and second principal components was 89.8%, containing the majority of the information on the three samples and presenting the primary features of the volatile aroma. The principal component analysis chart illustrates that the three sample groups were significantly separated, denoting the utilization of GC-IMS technology in the identification of the volatile compounds in the samples. When combined with PCA, the samples are better distinguished. Simultaneously, the findings reveal significant contrasts between the three samples.

### 3.5. Organoleptic Investigation

**Figure 7:**
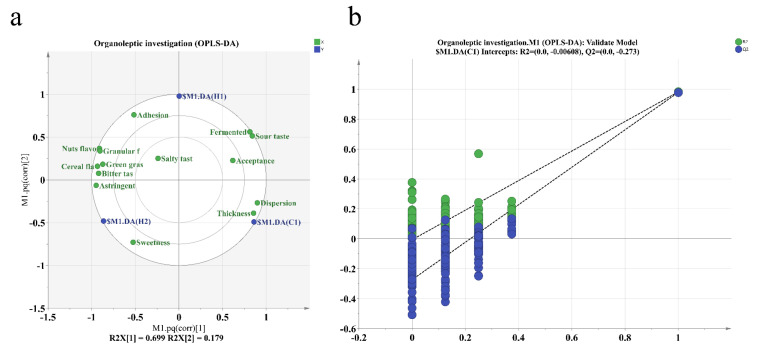
(**a**) OPLS-DA score plot and (**b**) OPLS-DA permutation test plot.

The sensory evaluations of three samples at varying levels of ripeness were analyzed using an OPLS-DA score plot. The results can be seen in Figure 7. The associated model was generated by randomly shuffling the order of categorical variables and yielded values of R2X = 0.699 and R2Y = 0.179. These R2 and Q2 values indicate a high level of general interpretation for the model when they fall within the range of 0.5 to 1 [51]. Q2 = 0.965 suggests the excellent predictive ability of the model. Clear stability and reliable predictability indicate that the model meets all the necessary requirements to identify the sensory differences of the three samples. To check for overfitting, we disrupted the categories of some samples and performed 200 instances of replacement fitting. Figure 5 demonstrates that R2 and Q2 intersect with the longitudinal axis at (0, −0.0068) and (0, −273), respectively. Additionally, the slopes of the two regression lines for R2 and Q2 are relatively high. The values for R2 and Q2 produced by the random arrangement on the left exhibit lower values compared to those on the right. Additionally, the fact that the Q2 regression line has a negative intercept suggests that the OPLS-DA model does not exhibit overfitting and demonstrates reliable predictive capabilities. This renders it suitable for discriminant analysis across diverse samples. Table 4 reveals that fermentation has significantly shifted the taste and flavor of walnut and purple rice yoghurt. The bitterness, sweetness, and astringency of H1 were significantly decreased (*p* < 0.05). In line with expectations, the fermented and sour tastes had the highest scores. Fermentation enhances food acidification, augmenting the sensory, nutritional, textural, and microbial safety of food. Furthermore, it negates the use of chemical preservatives and maintains the original natural state of the food. In our study, while fermentation significantly decreased the off-flavors of nuts and grains, it did not lower the unpleasant levels, which could be attributed to the new compounds generated by microbial fermentation [52]. Fermentation significantly enhanced the flavor, as well as the dispersibility and adhesion of the yoghurt, while reducing its graininess. The improved taste may be attributed to the fermentation process, which results in a smoother taste compared to the walnut and purple rice plant drink. The overall acceptance of the walnut and purple rice fermented plant drink is comparable to that of regular room-temperature yoghurt. Although the flavor of the walnut and purple rice fermented plant drink is slightly inferior to that of traditional yoghurt, its unique nutty and cereal notes add distinctive qualities. Moreover, team members rated it similarly to typical yoghurt in terms of degree of acceptance.

## 4. Conclusions

This study demonstrated the similarities and differences between the chemical, rheological, and organoleptic properties of a walnut–purple rice fermented plant beverage, walnut–purple rice fermented plant beverage, and room-temperature yoghurt. The results showed that the walnut–purple rice fermented plant beverage exhibited high pseudoplasticity and shear-thinning non-Newtonian fluid properties. Similar to the room-temperature yoghurt, the walnut–purple rice fermented plant beverage showed a semi-solid gelatinous structure with good viscosity and strong recovery properties. It is worth noting that the walnut–purple rice plant beverage before and after fermentation differed significantly from room-temperature yoghurt in terms of flavor and texture. Using an electronic nose, electronic tongue, GC-IMS, and sensory analysis, the walnut–purple rice fermented plant beverage was found to have a better taste. The results obtained in this study can show the potential of walnut and purple rice fermented plant beverages to be an alternative to animal-based yoghurt and can also provide a theoretical basis for the development of plant-based food products as well as the industrialization and further processing of walnut and purple rice.

## Figures and Tables

**Table 1:** Sensory word definitions and reference samples.

QDA	Description	Definition
Flavor	Green grass flavor	Odors associated with newly cut grass
	Nuts flavor	Odors associated with nuts
	Cereal flavor	Odors associated with cereals
	Fermented flavor	Acid substances produced by fermentation of dairy products, with an occasionally sour smell
Taste	Sweetness	Taste related to sucrose
	Sour taste	Taste associated with citric acid
	Bitter taste	Taste associated with caffeine
	Salty taste	Taste related to sodium chloride
	Astringent taste	The sample is placed on the tongue to make the tongue feel convergent
Taste	Dispersion	The extent of the sample in the mouth without chewing
	Thickness	The force, fluidity, and thinness of the tongue to suck the sample in the spoon into the mouth or expand the sample
	Adhesion	Mechanical texture characteristics related to the force required to move the adhesive object, stickiness
	Granular feeling	The number and size of particles that can be perceived in the sample
Overall	Acceptance	Acceptance of the sample

**Table 2:** Physicochemical analysis of the different samples.

	H1	H2	C1
Protein (%)	3.02 ± 0.04 ^a^	2.99 ± 0.04 ^b^	2.86 ± 0.02 ^c^
Fat (%)	6.67 ± 0.08 ^a^	6.02 ± 0.07 ^b^	2.93 ± 0.04 ^c^
Titration acidity	48.93 ± 1.70 ^b^	4.07 ± 0.68 ^c^	78.72 ± 1.41 ^a^
pH	4.45 ± 0.01 ^c^	6.38 ± 0.06 ^a^	4.54 ± 0.03 ^b^
Total solids (%)	29.09 ± 0.03 ^a^	28.74 ± 0.04 ^b^	20.55 ± 0.03 ^c^

H1: walnut and purple rice fermented plant drink, H2: walnut purple plant drink, C1: room-temperature yoghurt. Values are expressed as the mean ± standard deviation. Different lowercase letters indicate significant differences between groups for the same indicator (*p* < 0.05), the same as below.

**Table 3:** Results of volatile flavor components of samples.

Compound	Relative Retention Index	Relative Amount			Odor Description
		H1	H2	C1	
Aldehydes					
(−)-Perillaldehyde	1153.4	0.58 ± 0.01% ^b^	0.49 ± 0.03% ^b^	1.39 ± 0.04% ^a^	Perilla incense
(E, E)-2,4-heptadienal	1009.5	0.56 ± 0.06% ^a^	0.53 ± 0.031% ^a^	0.33 ± 0.03% ^a^	Melon and fruit fragrance
3-Methyl-2-butenal	762.6	2.94 ± 0.05% ^a^	1.52 ± 0.06% ^b^	1.83 ± 0.03% ^b^	Almond, roasted
Hexanal	1071.2	1.24 ± 0.01% ^b^	2.51 ± 0.08% ^a^	0.40 ± 0.03% ^c^	Apple, fat, fresh, green, oil
Pentanal-D	978.2	0.21 ± 0.00% ^b^	0.41 ± 0.01% ^a^	0.17 ± 0.00% ^c^	Grass smell
(E)-2-octenal	1070.2	0.61 ± 0.01% ^b^	0.70 ± 0.01% ^a^	0.18 ± 0.01% ^c^	Fat, fruit, grass, spice
Methional	906.1	0.82 ± 0.01% ^b^	1.53 ± 0.06% ^a^	0.38 ± 0.02% ^c^	Savory
Propionaldehyde	819	0.44 ± 0.01% ^b^	0.59 ± 0.02% ^a^	0.11 ± 0.00% ^c^	Floral, pungent, solvent
Pentanal-M	977	0.30 ± 0.01% ^ab^	0.25 ± 0.01% ^b^	0.39 ± 0.02% ^a^	Grass smell
Ketone					
Pentan-2-one	980	0.19 ± 0.03% ^a^	0.15 ± 0.01% ^c^	0.15 ± 0.06% ^b^	Flowers, fruit fragrance
Dihydro-2(3h)-furanone	908.7	0.51 ± 0.02% ^b^	0.82 ± 0.03% ^a^	0.26 ± 0.01% ^c^	-
Acetone	812.8	9.28 ± 0.02% ^a^	6.06 ± 0.10% ^b^	10.11 ± 0.12% ^a^	Pungent
2-Octanone	1304.7	3.42 ± 0.03% ^a^	2.97 ± 0.12% ^b^	0.78 ± 0.04% ^c^	Fat, fragrant, mold
3-Octanone	979.1	0.72 ± 0.02% ^a^	0.72 ± 0.06% ^a^	0.28 ± 0.02% ^b^	Butter, herbs, mold
Pent-1-en-3-ol	1156.3	1.39 ± 0.06% ^a^	1.40 ± 0.06% ^a^	1.05 ± 0.07% ^b^	Fruit fragrance, vegetable fragrance
1-Butanol	1116	0.36 ± 0.02% ^a^	0.37 ± 0.02% ^a^	0.35 ± 0.01% ^a^	Fruit
1-Propanol	1023.9	0.99 ± 0.02% ^a^	0.69 ± 0.02% ^b^	0.41 ± 0.02% ^c^	Alcohol, candy, pungent
1-Hexanol	873.6	0.23 ± 0.16% ^a^	0.63 ± 0.12% ^a^	0.33 ± 0.09% ^a^	Banana, flower, grass, herb
Ethanol	948.2	7.29 ± 0.01% ^a^	6.92 ± 0.03% ^a^	4.98 ± 0.02% ^b^	Alcohol, sweet
1-Pentanol	1242.4	0.35 ± 0.02% ^b^	0.29 ± 0.02% ^b^	0.89 ± 0.04% ^a^	Balsamic, fruit, pungent, yeast
2-Butanol	1008.8	0.64 ± 0.01% ^a^	0.10 ± 0.01% ^b^	0.15 ± 0.01% ^b^	Wine
3-Methyl-3-butenol	1245	0.72 ± 0.04% ^a^	0.78 ± 0.02% ^a^	0.62 ± 0.04% ^a^	-
2-Butoxyethanol	906.2	0.95 ± 0.12% ^a^	0.92 ± 0.04% ^a^	0.45 ± 0.03% ^b^	-
3-Heptanol	1304.1	0.40 ± 0.02% ^a^	0.33 ± 0.02% ^a^	0.15 ± 0.00% ^b^	Herbs
Esters					
2-Phenyl ethyl acetate	1240.9	1.27 ± 0.06% ^a^	1.17 ± 0.05% ^a^	4.90 ± 0.43% ^b^	Fruit
Ethyl benzoate	1155.4	1.80 ± 0.03% ^b^	1.29 ± 0.05% ^c^	3.64 ± 0.00% ^a^	Bitterness
Butyl-2-methylbutanoate	1041.3	0.40 ± 0.09% ^b^	0.70 ± 0.01% ^a^	0.83 ± 0.10% ^a^	Fruit fragrance
Ethyl acetate	871.5	0.38 ± 0.05% ^a^	0.43 ± 0.01% ^a^	0.31 ± 0.02% ^a^	Aromatic, brandy, grape
Methyl hexanoate	932.6	0.47 ± 0.09% ^a^	0.82 ± 0.08% ^a^	0.84 ± 0.22% ^a^	Pineapple
Ethyl pentanoate	902	3.03 ± 1.85% ^a^	6.61 ± 0.87% ^a^	3.92 ± 0.80% ^a^	Apple
n-Pentyl acetate	913.3	0.14 ± 0.06% ^a^	0.39 ± 0.06% ^a^	0.33 ± 0.10% ^a^	Pineapple, apple
Isopentyl formate	796.6	0.09 ± 0.00% ^b^	0.13 ± 0.01% ^a^	0.07 ± 0.00% ^b^	Apple
Iso-Propyl acetate	857.2	0.32 ± 0.02% ^a^	0.36 ± 0.02% ^a^	0.12 ± 0.00% ^b^	Banana
Methyl acetate	801.4	0.48 ± 0.01% ^b^	0.79 ± 0.02% ^a^	0.22 ± 0.02% ^c^	Mint, cool
Ethyl lactate	794.1	0.29 ± 0.01% ^b^	0.34 ± 0.00% ^a^	0.09 ± 0.00% ^c^	Cheese, floral, fruit, pungent
Ethyl 2-hydroxypropanoate	807.8	0.07 ± 0.00% ^b^	0.21 ± 0.00% ^a^	0.03 ± 0.00% ^c^	Rum, fruit, and creamy aroma
Ethyl trans-2-hexenoate	1045	6.42 ± 1.80% ^b^	12.46 ± 1.02% ^ab^	18.81 ± 3.49% ^a^	Fruit
Acids					
Acetic acid	1432	9.01 ± 0.55% ^a^	7.97 ± 0.03% ^ab^	6.71 ± 0.03% ^b^	Acid, fruit, pungent, sour
Cis-3-hexenyl lactate	1234.7	1.74 ± 0.19% ^c^	2.43 ± 0.03% ^b^	4.60 ± 0.35% ^a^	Green
Ethers					
Dibutyl sulfide	1069	2.61 ± 0.02% ^b^	3.68 ± 0.01% ^a^	0.45 ± 0.02% ^c^	Green
Dimethyl sulfide	770.2	2.11 ± 0.03% ^b^	3.78 ± 0.04% ^a^	0.90 ± 0.01% ^c^	Seafood flavor
Enolefins					
Undecane	1079.1	0.48 ± 0.01% ^a^	0.47 ± 0.01% ^b^	0.25 ± 0.02% ^b^	-
2,2,4,6,6-Pentamethylheptane	977.7	0.65 ± 0.01% ^b^	0.37 ± 0.03% ^c^	1.41 ± 0.05% ^a^	-
2,2,4,6,6-Pentamethylheptane-M	994	7.68 ± 0.06% ^a^	5.04 ± 0.25% ^b^	4.21 ± 0.02% ^c^	-
Beta-pinene	977	1.02 ± 0.01% ^ab^	1.15 ± 0.00% ^a^	0.88 ± 0.02% ^b^	Pine, polish, wood
o-Xylene	888.3	0.52 ± 0.16% ^a^	0.21 ± 0.03% ^a^	0.75 ± 0.12% ^a^	Fragrance
Acrylonitrile	1001.6	2.21 ± 0.01% ^a^	0.90 ± 0.01% ^b^	1.87 ± 0.01% ^b^	Peach kernel flavor
2-Acetyl-1-pyrroline	926.8	12.94 ± 0.31% ^a^	9.01 ± 0.35% ^b^	6.81 ± 0.33% ^b^	Savory
Styrene	866	0.08 ± 0.01% ^a^	0.09 ± 0.01% ^a^	0.14 ± 0.03% ^a^	Special aroma
Heterocyclic					
Tetrahydrofuran	887.2	0.61 ± 0.12% ^a^	0.50 ± 0.04% ^a^	1.06 ± 0.21% ^a^	Ethyl ether flavor
Methylpyrazine	1244.7	3.37 ± 0.03% ^b^	2.93 ± 0.05% ^b^	8.02 ± 0.00% ^a^	Nuts
2-Pentylfuran	1214.9	2.04 ± 0.07% ^a^	2.09 ± 0.09% ^a^	0.47 ± 0.04% ^b^	Butter, floral, fruit
Pyridine	1174.7	1.21 ± 0.07% ^a^	0.77 ± 0.01% ^b^	0.47 ± 0.00% ^c^	Odor
2-Acetyl-3-methylpyrazine	1074.1	0.30 ± 0.01% ^a^	0.18 ± 0.00% ^b^	0.11 ± 0.00% ^c^	Wood fragrance
2,5-Dimethylthiophene	1168.6	0.61 ± 0.02% ^a^	0.50 ± 0.01% ^b^	0.43 ± 0.00% ^b^	-

Values are expressed as the mean ± standard deviation. Different lowercase letters indicate significant differences between groups for the same indicator (*p* < 0.05). The odor descriptions are taken from the Odour Description Database (https://www.femaflavor.org/flavor-library and http://www.odour.org.uk (accessed on 1 October 2023)).

**Table 4:** Scores of 14 sensory attributes of three samples.

		**C1**	**H1**	**H2**
Flavor	Green grass flavor	1.25 ± 0.62 ^c^	3.42 ± 0.67 ^b^	4.50 ± 1.00 ^a^
	Nuts flavor	0.00 ± 0.00 ^c^	4.17 ± 0.72 ^b^	5.00 ± 0.74 ^a^
	Grainy flavor	0.00 ± 0.00 ^c^	2.17 ± 0.58 ^b^	3.33 ± 0.65 ^a^
	Fermented flavor	6.50 ± 0.80 ^b^	7.17 ± 0.58 ^a^	0.00 ± 0.00 ^c^
Taste	Sweetness	5.83 ± 0.58 ^b^	4.83 ± 0.72 ^c^	7.25 ± 0.45 ^a^
	Sour taste	5.83 ± 0.94 ^a^	5.92 ± 0.67 ^a^	0.00 ± 0.00 ^c^
	Bitter taste	0.33 ± 0.49 ^c^	1.92 ± 0.51 ^b^	3.17 ± 0.58 ^a^
	Salty taste	0.42 ± 0.51 ^b^	0.83 ± 0.39 ^a^	0.67 ± 0.49 ^ab^
	Astringent taste	0.25 ± 0.45 ^c^	1.75 ± 0.45 ^b^	3.75 ± 0.62 ^a^
Textural attributes	Dispersion	7.25 ± 0.45 ^a^	4.50 ± 0.52 ^b^	3.67 ± 0.78 ^c^
	Thickness	6.92 ± 0.51 ^a^	3.67 ± 0.49 ^b^	4.08 ± 0.67 ^b^
	Adhesion	4.08 ± 0.51 ^c^	6.50 ± 0.67 ^a^	5.50 ± 0.52 ^b^
	Granular feeling	0.00 ± 0.00 ^c^	4.00 ± 0.60 ^b^	4.83 ± 0.72 ^a^
Overall	Acceptance	7.17 ± 0.58 ^a^	6.92 ± 0.51 ^a^	6.08 ± 0.67 ^b^

^a–c^ Means within a row with different superscripts are different at *p* < 0.05.

## Data Availability

Data is contained within the article.
